# P-1298. Spatiotemporal Dynamics and Risk Factors of Scrub Typhus in 2013–2019 South Korea: a Bayesian Hurdle Model

**DOI:** 10.1093/ofid/ofae631.1479

**Published:** 2025-01-29

**Authors:** Jeehyun Kim, Byung Chul Chun

**Affiliations:** Korea University, Seoul, Seoul-t'ukpyolsi, Republic of Korea; Korea University College of Medicine, Seoul, Seoul-t'ukpyolsi, Republic of Korea

## Abstract

**Background:**

Scrub typhus (ST), also known as tsutsugamushi disease, is a common febrile vector-borne illness in South Korea, with rodents being the main host. Previous studies on vector-borne diseases have often lacked integrated spatiotemporal analyses covering disease dynamics, vectors, and environmental shifts. Therefore, we aimed to comprehensively explore high-risk areas and spatiotemporal trends of ST, incorporating vectors and environmental information.

**Figure 1.**
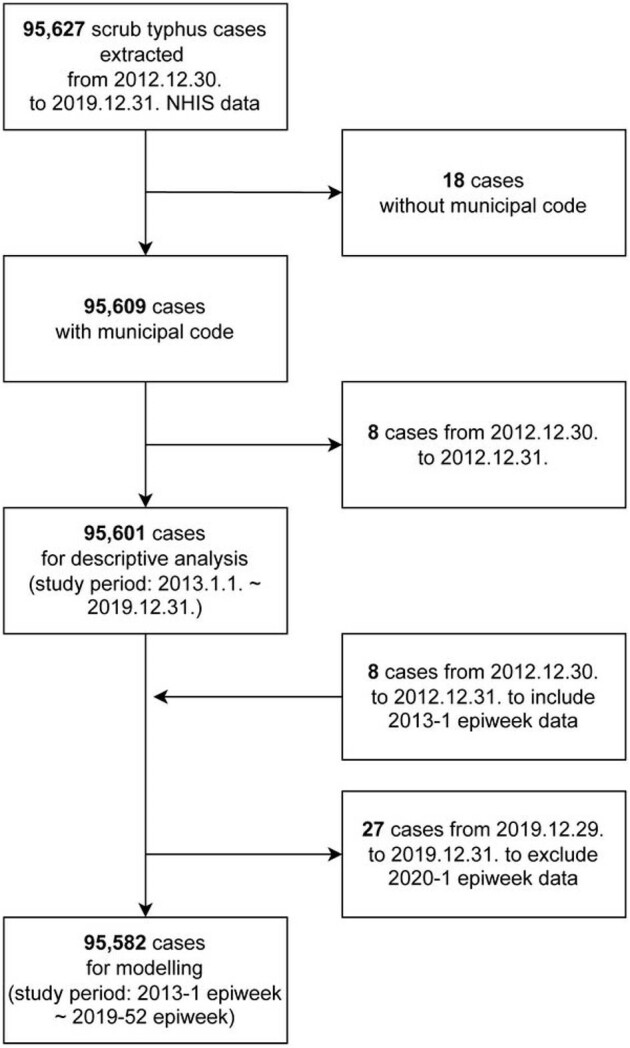
Flowchart of study subject selection. Scrub typhus was diagnosed with A75.3 in ICD-10-CM Code.

Epiweek, epidemiological week. NHIS, National Health Insurance Service in South Korea.

**Methods:**

ST cases were extracted from the 2013–2019 Korea National Health Insurance Service data at 250 municipal levels. Spatial and temporal clusters were assessed using Getis-Ord G_i_* and Hot and cold spot trend analyses. Bayesian hurdle models with spatiotemporal interaction terms were employed to identify associations between ST incidence and diverse regional factors, encompassing rodent suitability, as well as human and environmental factors. Stratification by gender and age group (0–19, 20–39, 40–59, 60–79, and ≥80 years) was performed.

**Figure 2.**
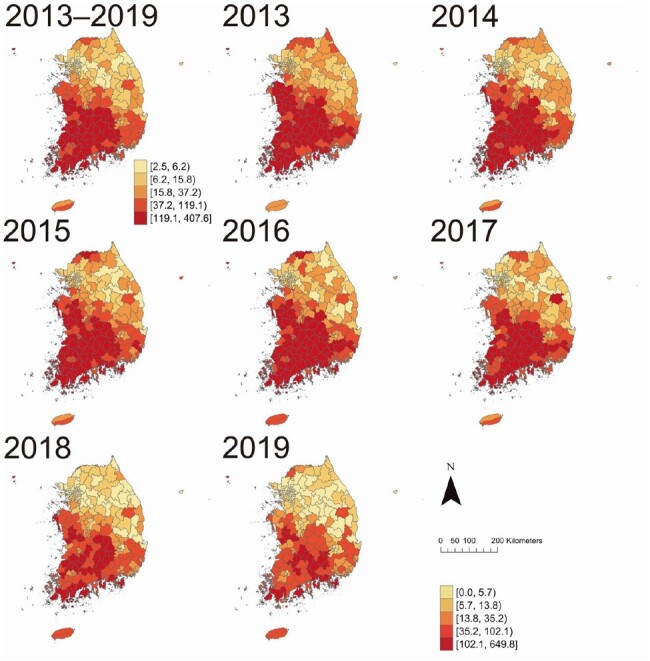
Scrub typhus incidence per 100,000 population by year.

**Results:**

Between 2013 and 2019, 95,601 ST patients were reported (male-to-female incidence ratio=0.71:1). ST incidence had positive spatial autocorrelation across South Korea (*I*=0.600, p=0.01), with spatial expansion from southwestern to northeastern regions. Municipalities with higher rodent suitability (coefficient=0.230, 95% credible interval=0.126–0.333), forest area (coefficient=0.254, 95% credible interval=0.117–0.394), and worse financial independence (coefficient=-0.262, 95% credible interval=-0.326 to -0.198) had higher likelihoods of increased ST incidence, even after adjusting for spatiotemporal autocorrelation. However, significant risk factors varied by age group.

**Figure 3.**
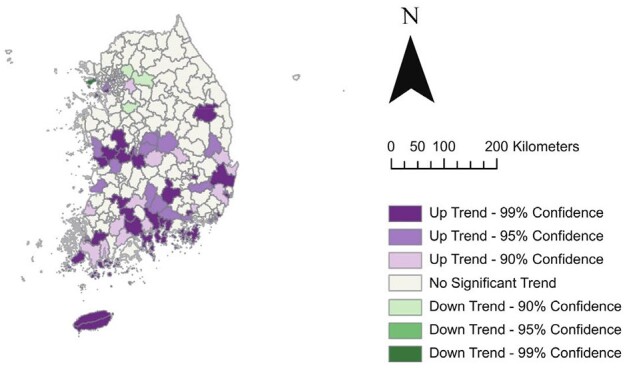
Results of hot and cold spot trends by visualizing a space-time cube of scrub typhus incidence per 100,000 population. Colors represent the patterns of hot and cold spot trends, classified as “up trend,” “down trend," or “no significant trend” with 90, 95, and 99% confidence levels.

**Conclusion:**

We elucidated the intricate spatiotemporal dynamics of scrub typhus in South Korea, accounting for factors such as rodent suitability, forest coverage, and economic independence, even after adjusting for spatiotemporal autocorrelation. Our findings lay the groundwork for evidence-based strategies and policies to tackle scrub typhus, integrating a holistic One Health perspective and emphasizing rodent distribution as a key determinant of disease risk.

**Table.**
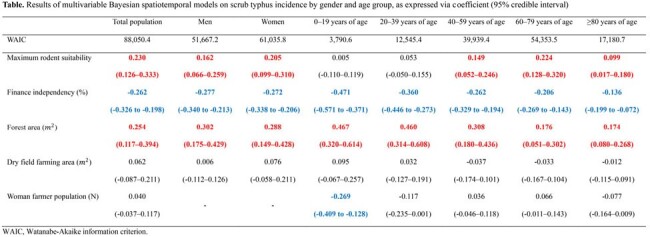
Results of multivariable Bayesian spatiotemporal models on scrub typhus incidence by gender and age group, as expressed via coefficient (95% credible interval) WAIC, Watanabe-Akaike information criterion.

**Disclosures:**

**All Authors**: No reported disclosures

